# Transcriptional responses of *Daphnis nerii* larval midgut to oral infection by *Daphnis nerii* cypovirus-23

**DOI:** 10.1186/s12985-021-01721-x

**Published:** 2021-12-14

**Authors:** Wendong Kuang, Chenghua Yan, Zhigao Zhan, Limei Guan, Jinchang Wang, Junhui Chen, Jianghuai Li, Guangqiang Ma, Xi Zhou, Liang Jin

**Affiliations:** 1 Institute of Microbiology, Jiangxi Academy of Sciences, No. 7777 Changdong Road, Nanchang, 330096 China; 2 State Key Laboratory of Virology, Wuhan Institute of Virology, Chinese Academy of Sciences (CAS), Wuhan, 430071 China; 3 School of Life Sciences, Jiangxi University of Traditional Chinese Medicine, Nanchang, 330004 China

**Keywords:** *Daphnis nerii* cypovirus-23, Midgut, Transcriptome analysis

## Abstract

**Background:**

*Daphnis nerii* cypovirus-23 (DnCPV-23) is a new type of cypovirus and has a lethal effect on the oleander hawk moth, *Daphnis nerii* which feeds on leave of *Oleander and Catharanthus* et al. After DnCPV-23 infection, the change of *Daphnis nerii* responses has not been reported.

**Methods:**

To better understand the pathogenic mechanism of DnCPV-23 infection, 3rd-instar *Daphnis nerii* larvae were orally infected with DnCPV-23 occlusion bodies and the transcriptional responses of the *Daphnis nerii* midgut were analyzed 72 h post-infection using RNA-seq.

**Results:**

The results showed that 1979 differentially expressed *Daphnis nerii* transcripts in the infected midgut had been identified. KEGG analysis showed that protein digestion and absorption, Toll and Imd signaling pathway were down-regulated. Based on the result, we speculated that food digestion and absorption in insect midgut might be impaired after virus infection. In addition, the down-regulation of the immune response may make *D. nerii* more susceptible to bacterial infections. Glycerophospholipid metabolism and xenobiotics metabolism were up-regulated. These two types of pathways may affect the viral replication and xenobiotic detoxification of insect, respectively.

**Conclusion:**

These results may facilitate a better understanding of the changes in *Daphnis nerii* metabolism during cypovirus infection and serve as a basis for future research on the molecular mechanism of DnCPV-23 invasion.

**Supplementary Information:**

The online version contains supplementary material available at 10.1186/s12985-021-01721-x.

## Introduction

The oleander hawk moth, *Daphnis nerii* (*D. nerii*), belongs to Lepidoptera, Sphingidae family, is a worldwide pest [1]. *D. nerii* larvae damages leave of Oleander, *Catharanthus, Vinca, Adenium, Vitis, Tabernaemontana, Gardenia, Trachelospermum, Amsonia, Asclepias, Carissa, Rhazya, Thevetia, Jasminum and Ipomoea* [2, 3], which affect the landscape and the medicinal value of these plants. At present, the chemical pesticide decamethrin is used to control *D. nerii* [2].

*Cypovirus* is a member of the *Reoviridae* family, and is characterized by its single layered capsid [4]. DnCPV-23 was isolated from naturally diseased *D. nerii* larvae. This was a new type of cypovirus based on different electrophoretic migration patterns and conserved terminal sequences [1, 5, 6]. In addition to *Daphnis nerii*, it has been found that DnCPV-23 can also induce infection and death in many species of Sphingidae insects, such as *Cephonodes hylas* Linnaeus, *Ampelophaga rubiginosa* Bremer & Grey, and *Agathia lycaenaria* Kollar. The genome of DnCPV-23 consists of ten segments of linear double-stranded RNA, referred to as genomic segments 1 *(S1*) to 10 (*S10*), in accordance with the fragments from longest to shortest [7]. Our previous research and unpublished data demonstrated that the virus could successfully replicate on the Sf9 [8] and Manduca sexta cell lines QB-MS 2-2 [9]. However, the molecular mechanism of the interactions between the new type cypovirus and its hosts remains unclear. It is necessary to identify the interactions between the virus and its hosts to achieve an in-depth understanding and reveal the exploitation potential of the virus for future insecticide development.

Recently, many studies in the field have generated large amounts of data using the aforementioned high-throughput approaches, from the silkworms or BmN cells infected with BmCPV, including (1) The possible host’s RNAi response against BmCPV challenge in persistent and pathogenic Bombyx mori model was compared. During the pathogenic infection, it was found that higher level RNAi responses against BmCPV were observed, which further demonstrated the importance of RNAi as an antiviral mechanism [10]. (2) Gene expression profiles [11–19], DNA methylation [20], and lipidomic profile [21] of silkworm midgut or BmN cells after BmCPV infection were analyzed. These results suggested that many genes (for example, genes expressing Calreticulin, FK506-binding protein, and protein kinase c inhibitor gene, microRNAs, and activated protein kinase C) may play important roles in BmCPV replication. In addition, epigenetic regulation may influence silkworm-virus interaction, and BmCPV may modulate the lipid metabolism of cells for their self-interest.

Until now, the molecular mechanism underlying the midgut infection of DnCPV-23 is not clearly understood. Furthermore, since transcriptome analyses regarding *D. nerii* or DnCPV-23 have not yet been performed, this study aims to fill this gap about the new type cypovirus. The data and analysis presented here provide insights into the possible mechanism of DnCPV-23 infection and host defense and a basis for future DnCPV-23 relevant studies.

## Materials and methods

### *Daphnis nerii* larval midgut and virus stock

Newly wild-caught second instar larvae with a similar mass were used in this research investigation for the virus infection. Before infection, the *D. nerii* were supplied with 12-h day/night cycles under 50 ± 5% relative humidity conditions and were nurtured on oleander leaves at 27 ± 1 ℃ for three days. The midgut tissues were collected from four pathogenically infected larvae at 72 h [13, 15] after feeding with DnCPV-23. The same tissues were also collected from three uninfected control larvae at the same time point. DnCPV was originally isolated from the larvae of *D. nerii* and propagated in *D. nerii* larvae [1]. The polyhedra suspension of DnCPV-23 utilized for infecting the *D. nerii* was stored at 4 °C in the dark.

### Virus inoculation

In this study, the DnCPV-23 viral stock was suspended in distilled water at a concentration of 2 × 10^7^ polyhedra/mL. Then, 100 μL of the viral suspension was spread evenly on one piece of oleander leaf measuring approximately 4 cm × 1.5 cm each in size. The leaf was then fed to four *D. nerii* larvae. The dose of infection was calculated as 2 × 10^6^ polyhedra per larva. In addition, three control larvae were fed the same quantity of leaves treated with only distilled water. After approximately 12 h, fresh oleander leaves were used to feed the inoculated larvae after the DnCPV-23-inoculated leaves had been completely consumed.

### Sample preparation

The midguts of both DnCPV-23-infected and control larvae were collected at 72 h post-inoculation by dissecting the larvae on ice. The isolated midgut was then quickly washed in 0.8% diethylpyrocarbonate (DEPC)-treated physiologic saline solution to remove the attached leaf pieces, and then frozen in liquid nitrogen [13, 22].

### RNA sequencing

All of the RNA-seq procedures were conducted by the Oebiotech Company (Shanghai, China). The total RNA was extracted from the *D. nerii* midgut tissue using TRIzol reagent (Invitrogen, USA) according to the manufacture’s protocols. The RNA integrity and concentrations were checked using an Agilent 2100 Bioanalyzer (Agilent Technologies, USA). In addition, seven RNA samples (including three uninfected samples and four infected samples) with RNA integrity were used to construct the libraries. The cDNA libraries were prepared using a TruSeq RNA Sample Preparation Kit (Illumina, USA) according to the manufacturer’s protocols. Thereafter, the obtained cDNA libraries were sequenced on the Illumina HiSeq2500 platform, which generated paired-end raw reads of 125 bp.

### De novo assembly and functional annotation

The raw data was pretreated by discarding reads with adaptors and low quality (quality scores < 30). Then, the raw data was assembled using Trinity software with default parameters for de novo transcriptome assembly. Transcripts that were not shorter than 300 bp were used for subsequent analysis. To obtain the functional annotations of predicted protein-coding sequences, we searched against various databases, including the NCBI non-redundant (NR) protein, SwissProt, and euKaryotic Orthologous Groups (KOG) using Blastx with an E-value < 10^−5^. The top hit was utilized to assign gene names. Whereafter, the Gene Ontology (GO) annotations of the transcripts were then analyzed based on SwissProt annotations, and functional classifications were assigned by WeGO software. In addition, for the purpose of determining the biological pathways involved, the KEGG pathway was annotated based on the KEGG Orthology (KO) identifiers.

### Differential gene expression analysis

RNA sequencing results from the two groups were mapped to the assembled transcriptome using bowtie2 [23] and express [24]. The FPKM (fragments per kb per million reads) method [25] was utilized to calculate the expression levels of the unigenes, which eliminated the influencing effects of the different gene lengths and sequencing levels. The differences in the unigene expressions between the two groups were calculated with DESeq [26] and any significant differences were determined with *P* < 0.05 and an absolute value of log2 fold change > 1.

### Real-time quantitative reverse transcription PCR (Real-Time qRT-PCR)

This study utilized qRT-PCR to analyze the expression level of DnCPV-23 *S1*, *S10* genes of transcriptome samples, and verify the DEGs recognized by the RNA-seq. The total RNA was isolated from the samples of the transcriptomic analysis using TRIzol reagent (Life Technologies) and was then treated with DNase I (Fermentas, Glen Burnie, MD, USA). We reversely transcribed 1 μg of the total RNA per sample into complementary DNA (cDNA) using a PrimeScript RT Reagent Kit (Takara). Then, qRT-PCR was performed using Talent qPCR PreMix SYBR Green (Tiangen, China) on a QuantStudio™ 7 Flex Real-Time PCR System (Applied Biosystems™). One cycle was added for melting curve analysis for all the reactions to verify the product specificity. The expression level of each gene relative to that of the *RPL13* gene was calculated using the 2^−△△CT^ method [27]. All of the primers for the aforementioned target genes are listed in Table 1. Results are representative of two to three independent experiments.

**Table 1:** Primers used in the qRT-PCR for the the viral RNA detection of transcriptome samples and validation of the RNA-seq

No	Primer name	Primer sequence (5' to 3')	Tm (°C)	Gene id	Target gene
	S1-RTPCR-F	GTGCTGATGGTCTGCTAA	49.6	N/A	DnCPV *S1*
	S1-RTPCR-R	TGATTGATGACGACATTGAG	51.5		
	S10-RTPCR-F	GTCCGCCAATACTCTCAG	52.6	N/A	DnCPV *S10*
	S10-RTPCR-R	CGTAGTCCATCGTCAATCA	51.3		
1	CASP8-F	ACTGGAGAAGACTATGAGGTTA	51.5	TRINITY_DN10280_c0_g1_i1_3	*CASP8*
2	CASP8-R	ACGCTGTCATCTTGGCTAA	53.7		
3	CYP6AB13-F	GATTCACACCAGCATTCAG	51.0	TRINITY_DN11437_c0_g1_i1_6	*CYP6AB13*
4	CYP6AB13-R	CAGTCGTATATCTCGCCATA	50.5		
5	CYP6B45-F	GCGATACCGAACCAGAAC	53.4	TRINITY_DN12532_c0_g7_i1_1	*CYP6B45*
6	CYP6B45-R	ATTGGCAGTAAGTGTGAGTT	51.0		
7	DHRS4-F	TCTTCTATCGCCGCATATCA	52.8	TRINITY_DN12896_c1_g2_i3_3	*DHRS4*
8	DHRS4-R	CACCACCTCATTAGCAATCG	53.5		
9	PNLIP-F	CACCTCGTAGACTTGGAAGA	53.5	TRINITY_DN12381_c0_g2_i1_6	*PNLIP*
10	PNLIP-R	GTTAGCGTTGCCATTGACA	53.2		
11	PRSS1_2_3-F	CCTGGAAGATGGCGTGTT	55.4	TRINITY_DN10836_c0_g5_i1_6	*PRSS1_2_3*
12	PRSS1_2_3-R	TCGGCGGTAATTCGGTTAT	53.5		
13	RDH12-F	GTCTAATCGTCCGCTATTGAG	52.5	TRINITY_DN14445_c0_g1_i1_3	*RDH12*
14	RDH12-R	CTGTAGGTGAAGATTGCCATT	52.2		
15	SCARB1-F	AACACAACAAGAGGCATCAC	53.0	TRINITY_DN14140_c0_g1_i1_6	*SCARB1*
16	SCARB1-R	GTCGTCGGTTCAATATCCATAA	51.7		
17	SLC46A1-F	TGGAACGACACGACAAGT	53.7	TRINITY_DN8071_c0_g1_i2_5	*SLC46A1*
18	SLC46A1-R	CAACAGAGTGCGAACAGTATA	51.7		
19	SLC52A3-F	AAGCGATTGTGGAAGATGTC	52.5	TRINITY_DN11521_c0_g1_i2_4	*SLC52A3*
20	SLC52A3-R	CGGCATACACGAGTACGA	54.4		
21	ABCA3-F	CGATATACGCCGCAAGTAAG	53.3	TRINITY_DN12365_c0_g1_i6_2	*ABCA3*
22	ABCA3-R	GCAGTTCTCTACATTCAGTTGA	51.8		
23	ABCC4-F	AGTGGATGGAAGGTTGGAAT	53.3	TRINITY_DN11997_c1_g1_i24_2	*ABCC4*
24	ABCC4-R	CGGCTCTTGTGGTATAATTGA	51.9		
25	CYP6B6-F	GGACTATTGTTGGCGAATC	50.7	TRINITY_DN13898_c0_g1_i1_4	*CYP6B6*
26	CYP6B6-R	TTGTGGAAGAAGACGATGT	50.5		
	GAPDH-F	TATGTTCGTTGTCGGAGTTA	50.1	TRINITY_DN5984_c0_g1_i2_2	*GAPDH*
	GAPDH-R	TAGCAGTAGTGGCGTGTA	52.4		
27	LYPLA3-F	ACATCCACGACACAAGACTA	52.8	TRINITY_DN10250_c0_g1_i1_1	*LYPLA3*
28	LYPLA3-R	GACCGATAATGAACTCCTGAAT	51.5		
29	NTE-F	CAGCCTGGAAGGTAAGTAGT	53.6	TRINITY_DN14343_c0_g2_i1_4	*NTE*
30	NTE-R	CTCATAGACGAGCGACAGT	53.8		
31	UGT-F	GCATTCATTCAAGTCCATCAG	51.3	TRINITY_DN14215_c0_g5_i7_5	*UGT*
32	UGT-R	GCCTCCATCAATAATCACCAA	52.2		
33	DnRPL13-F	GAACTATTGGCATTGCTGTTG	52	TRINITY_DN4717_c0_g1_i2_3	*RPL13*
34	DnRPL13-R	TCCTCCTCATTGGCTTCAC	54.5		

## Results

### Virus infection of the samples

**Fig. 1:**
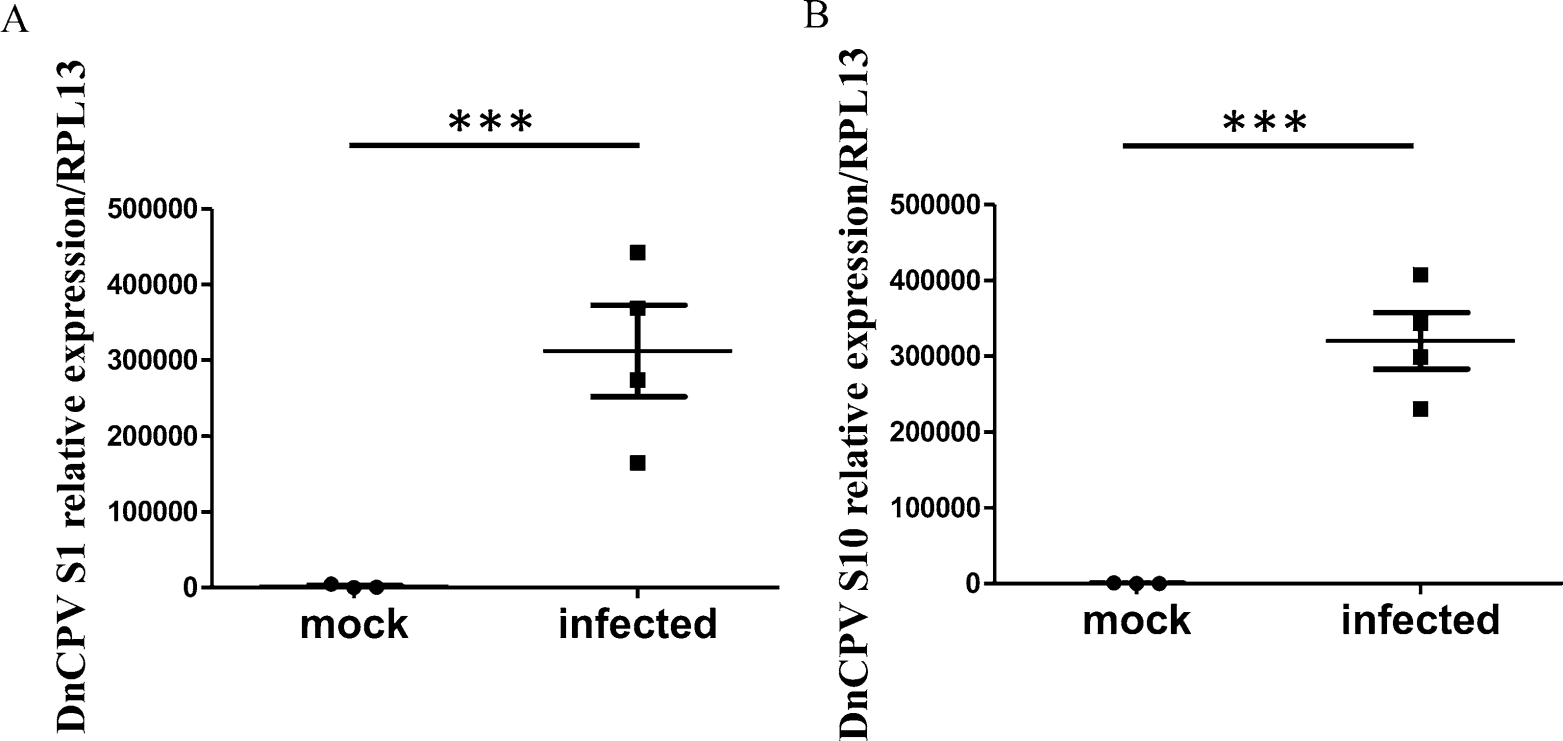
Detection of the viral RNA in transcriptome samples at 72 hpi (hours post infection). After feeding for 72 h, the mRNA levels of DnCPV-23 *S1* (**A**) and *S10* (**B**) in the midgut of *D. nerii* were detected. The asterisk (***) denotes the presence of a statistically significant difference (*p* < 0.001) by unpaired Student's t test

Prior to the transcriptome analysis, qRT-PCR was used to detect the mRNA levels of the DnCPV-23 *S1* and *S10* genes in the infected and uninfected samples. The results showed that the infected group had been successfully infected based on the high relative expression of the viral gene mRNA compared with uninfected group (Fig. 1).

### Transcriptome sequencing and assembly

The RNA-Seq data from the DnCPV-23-infected and control groups contained 346.39 million reads, and 334.60 million clean reads after trimming, among which 96.17 to 97.39% per sample were determined to be useful. The acquired clean reads were assembled into 31,696 unigenes (> 300 bp). The average length of these unigenes was 1347.61 bp, and the N50 length was 2348 bp; other information about these unigenes were shown in Table 2. This study then assembled 31.696 unigenes ranging from 301 bp to 32,420 bp. The total unigene length was 42,713,980.

**Table 2:** Statistics of the assembly results

Term	All	> = 500 bp	> = 1000 bp	N50	Total_Length	Max_Length	Min_Length	Average_Length
Unigene	31,696	20,703	12,663	2348	42,713,980	32,420	301	1347.61

### Transcriptome annotation

**Fig. 2:**
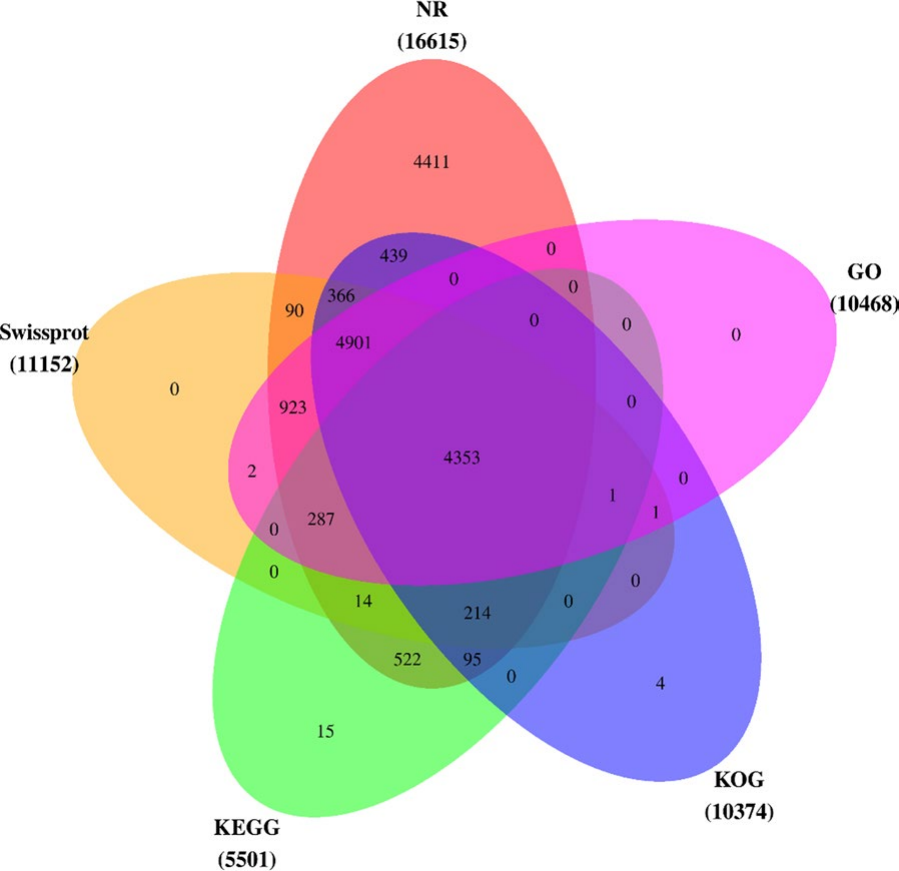
Venn diagram showing the degree of overlapping of the unigenes annotated based on different databases. Numbers in different colors represent the number of unigenes annotated through one or more annotation libraries

A total of 31,696 assembled unigenes were searched against the public databases, including the NR, Swissprot, KOG, GO, and KEGG databases, among which 16,820 (53.1%) (Fig. 2) unigenes were annotated. The distribution patterns of the unigenes in the different databases were as follows: 16,615 unigenes in the NR database, 11,152 unigenes in the Swissprot database, 10,374 unigenes in the KOG, 10,468 unigenes in the GO, and 5501 unigenes in the KEGG databases (Table 3). Figure 2 shows the degree of overlap between the unigenes annotated in the different databases. It was found that 4353 (13.7%) unigenes overlapped in all five databases, while 12,390 (73.7%) unigenes overlapped in two or more databases.

**Table 3:** Annotation statistics for each database

Anno_Database	Annotated_Number	300 < = length < 1000	Length > = 1000
NR	16,615(52.42%)	6217(19.61%)	10,398(32.81%)
Swissprot	11,152(35.18%)	2921(9.22%)	8231(25.97%)
KEGG	5501(17.36%)	1694(5.34%)	3807(12.01%)
KOG	10,374(32.73%)	2758(8.70%)	7616(24.03%)
eggNOG	15,249(48.11%)	5239(16.53%)	10,010(31.58%)
GO	10,468(33.03%)	2670(8.42%)	7798(24.60%)
Pfam	10,594(33.42%)	2505(7.90%)	8089(25.52%)

### Significant impacts of the viral infection on the hosts’ transcriptome expressions

**Fig. 3:**
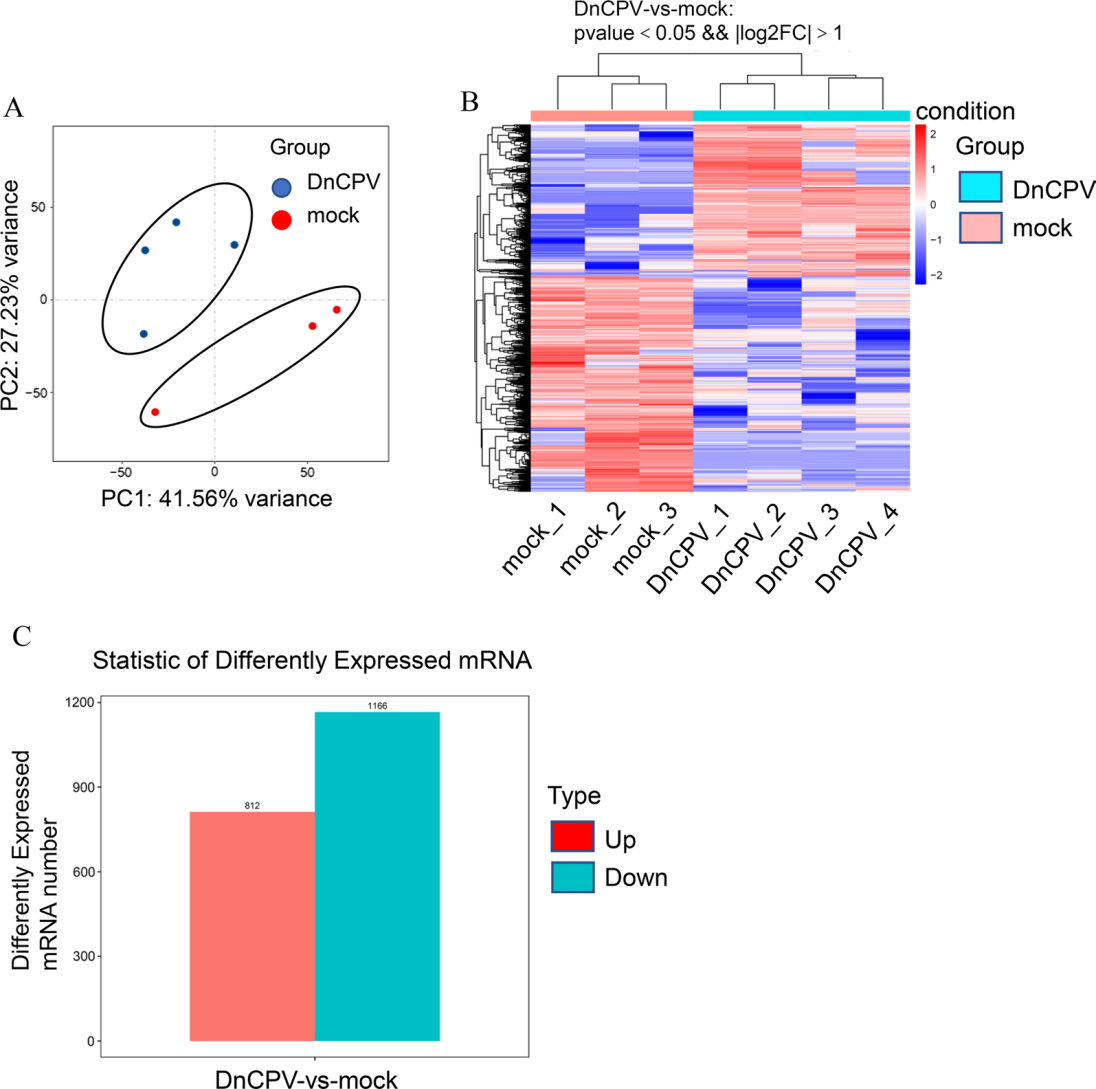
Influence of DnCPV-23 infection on D. *nerii* transcriptome: **A** Plot of the 1st and 2nd principal component of the sample variations using the principal component analysis, in which the red dots represent samples without DnCPV-23 infection, and the green dots denote infected samples. **B** Heat map of 1,978 differently expressed genes (DEGs) in the infected samples and controls. **C** After infection, 812 genes were up-regulated (red bars) and 1166 genes were down-regulated (blue bars)

As shown in Fig. 3, the main component PCA1 had reached 41.56%, and the main component PCA2 had reached 27.23%. Therefore, the percentage total of the two was 68.79%, which accounted for a high proportion and represented the overall population to a large extent. This study’s principal component analysis manifested a clear separation of the samples with the two treatments (Fig. 3A), which indicated that the samples had good repeatability. The heat map of the gene expressions is presented in Fig. 3B. The results suggested that these DEGs could distinguish the samples. The results revealed that the viral infection could exert apparent influences on the midgut gene expressions. In addition, the transcriptome results showed that 1166 genes were down-regulated (accounting for 3.68% of the total assembled unigenes) and 812 genes (accounting for 2.56% of the total assembled unigenes) were up-regulated as a response to the DnCPV-23 infection (Fig. 3C).

### Analysis of the differently expressed genes

**Fig. 4:**
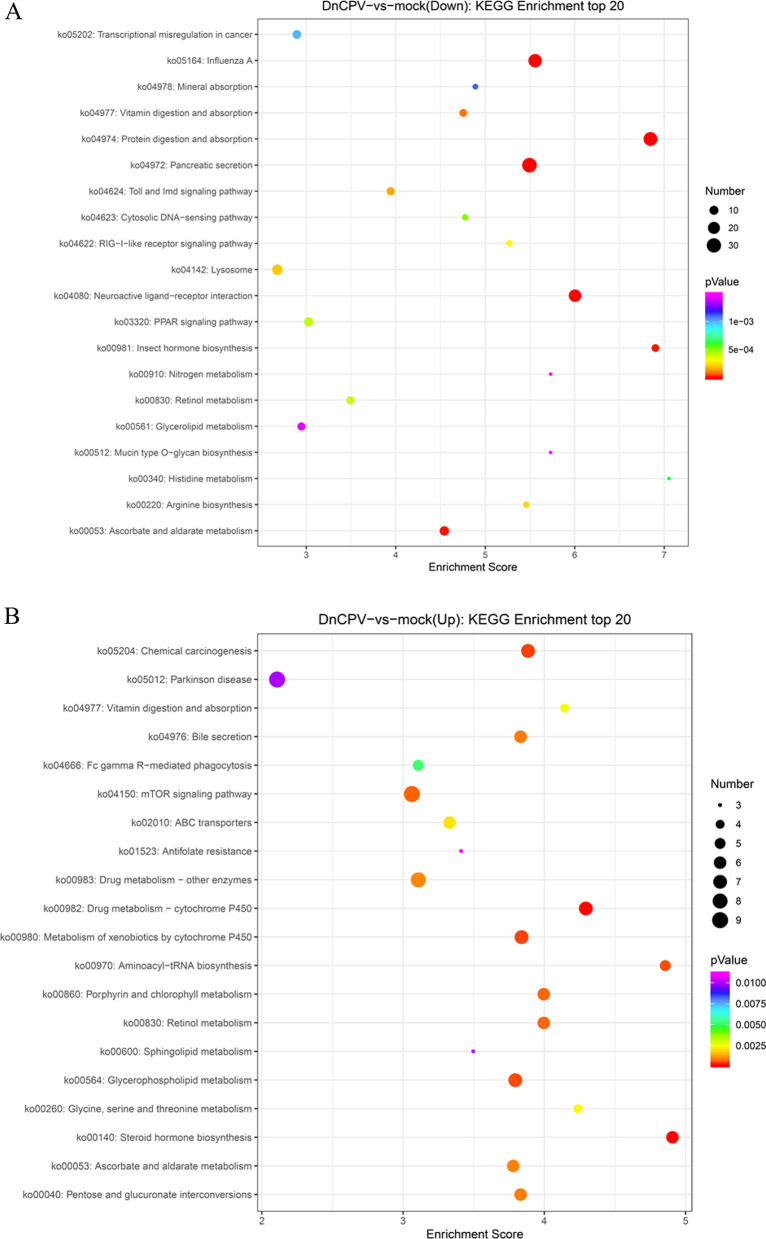
KEGG classifications of DEGs after DnCPV-23 infection (Top 20): **A**. Down-regulated pathways; **B**. Up-regulated pathways. Horizontal axis of the figure is the enrichment score. The larger the bubble, the more the number of DEGs. The bubble color changes from purpl E-blu E-green–red, indicating that the smaller the enrichment pValue and the greater the significance

In this study, KEGG function enrichment analysis was performed on the differential genes expressed in the DnCPV-23-infected and uninfected control groups to clarify the relevant biological pathways involved in the differential genes. Among all of the DEGs, 298 DEGs had KEGG annotations, of which 118 were up-regulated genes and 180 were down-regulated genes. According to the pValue of KEGG analysis of up-regulated and down-regulated signal pathways, we identified 20 most significant signal pathways each. These pathways play an important role in insect reproduction, immunity, digestion and absorption and xenobiotic metabolism and so on (Fig. 4).

### qRT-PCR validation of DEGs

**Fig. 5:**
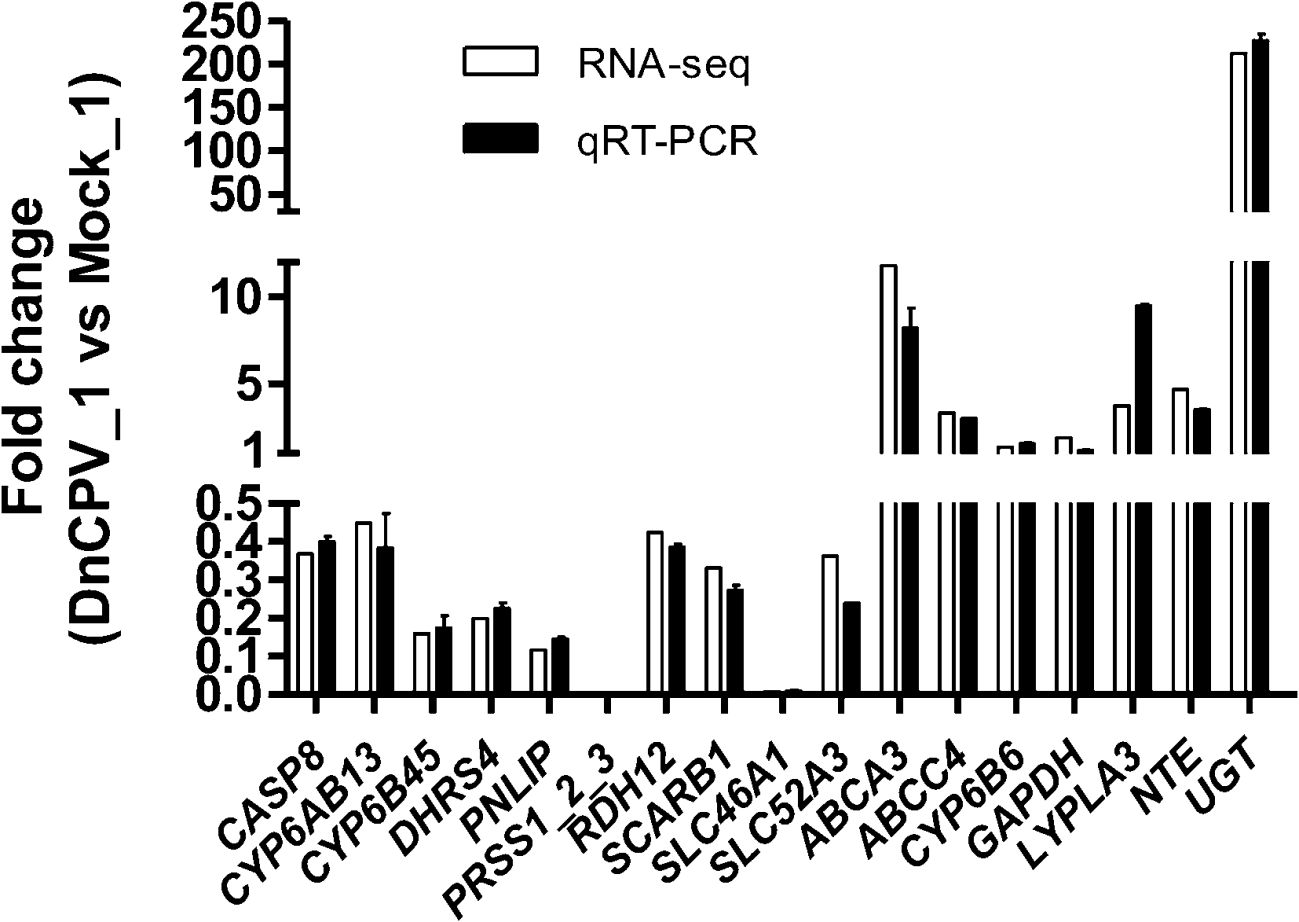
Validation of RNA-seq profiles by real-time qPCR. To validate the RNA-seq data, the relative mRNA levels of 17 selected DEGs in the DnCPV_1 sample were examined by qPCR; The mRNA levels by qPCR are presented as the fold change compared with the Mock_1 sample after normalization against *RPL13*. The relative expression levels from the RNA-seq analysis were calculated as RPKM values. Error bars show mean ± SEM

To verify the reliability of the transcriptome data and the DEG results obtained by RNA-seq, seventeen DEGs were selected for qPCR analysis. As shown in Fig. 5, the fold-change values of DnCPV_1 sample vs Mock_1 sample obtained in the qPCR analysis results were consistent with the values obtained by the RNA-seq for all of the selected genes.

## Discussion

This study analyzed the transcriptome of the uninfected *D. nerii* midgut and the DnCPV-23- infected *D. nerii* midgut presented unique gene expression profiles induced by DnCPV-23 infection for the first time. In addition, KEGG function enrichment analysis was performed on the differential genes expressed after DnCPV-23 infection. Compared with uninfected D. *nerii* midgut, the transcriptome profiles of the infected samples displayed universally changed transcript abundances for many pathways.

Based on the pValue of KEGG analysis regarding up-regulated and down-regulated signal pathways, we identified 20 most significant signal pathways each. Among these signal pathways, the retinol metabolism pathway, vitamin digestion, and absorption signal pathway were down-regulated, consistent with the transcriptome study about BmCPV infected midgut vs non-infected midgut [13]. In addition, protein digestion and absorption pathway way was down-regulated in accord with previous research [10]. DnCPV infection may destroy the functions of digestion and the absorption of midguts, which causes the disturbance of protein and amino acid metabolism in *D. nerii* [13, 28]. Peptidoglycan recognition proteins (PGRPs) are pattern recognition molecules that are conserved from insects to mammals. PGRPs are the first receptors known to recognize, bind, or catalytically cleave the pathogenic microorganisms [29], PGRPs recognize bacteria and their unique cell wall component, eptidoglycan [30, 31]. This study observed nine transcripts of *D. nerii* isoforms of *PGRP* genes. Six transcripts were found to be down-regulated in the infected *D. nerii* midgut. The most highly expressed and most dramatically down-regulated was TRINITY_DN13195_c0_g1_i3_3, which was down-regulated by as much as 51-fold. The down-regulation of *PGRP* expression can lead to a decrease in the ability of the *D. nerii*’s innate immune system to recognize bacterial peptidoglycans (PGN), which may lead to *D. nerii* more susceptible to bacterial infections. In addition, *BmPGRP-S2* was up-regulated upon BmCPV infection, overexpression of which can activate the Imd pathway and induce increased AMPs to enhance the antiviral capacity of transgenic silkworm against BmCPV [32]. Moreover, previous study demonstrates [33] that PGRPS2-1 and PGRPS2-2 can prevent BmCPV replication. Based on this work, was speculated that the down-regulation of *PGRP* was conducive to the replication of DnCPV-23.The gene *CASP8* (KEGG gene name: caspase-8, Gene id: TRINITY_DN10280_c0_g1_i1_3) (Dredd in Drosophila) was down-regulated more than two folds, and other caspase genes changed non-significantly. It is predicted to be involved in the cleavage of Relish, the Drosophila homolog of mammalian NF-κB, resulting in activating the immune-deficient pathway (IMD)-induced expression of antimicrobial peptides in response to Gram-negative bacteria [34–36], fungi and viruses [37]. Research performed by Li et al. proved BmDredd interacts with BmSTING to enhance antiviral signaling [38]. The down-regulation of this gene may be very important for DnCPV-23 to escape from the host innate immune system and replicate in the midgut. Our result conflicted with the work by Guo et al. [11]. We speculated the contradiction might be related to the different stages of virus-host interaction or the heterogeneity of different species against virues. The pathways and the genes mentioned above are listed in Table 4 (The expression of genes in each sample is shown in Additional file 1).

**Table 4:** The down-regulated pathways focused in the discussion section

id	Term	pValue	Enrichment_score	gene_id	BaseMean_control_mock	BaseMean_case_DnCPV	FoldChange	pValue	qValue	Regulation	NR annotation	KEGG gene name
ko04974	Protein digestion and absorption	1.33E−17	6.845688889	TRINITY_DN12884_c1_g5_i1_1	2843.901036	282.1111777	0.099198662	1.72 E−06	0.0007668	Down	LOW QUALITY PROTEIN: carboxypeptidase B [Bombyx mori]	CPA2
				TRINITY_DN13745_c3_g2_i2_4	37,228.13358	6283.238748	0.168776625	0.04653249	0.7667028	Down	putative chymotrypsin, partial [Samia ricini]	CELA2
				TRINITY_DN13546_c1_g2_i2_1	33,763.67895	179.8629852	0.005327115	1.26 E−10	2.06 E−07	Down	RecName: Full = Trypsin, alkaline C; Flags: Precursor	PRSS1_2_3
				TRINITY_DN11633_c0_g1_i2_3	576.0371804	28.38155477	0.049270352	1.62 E−08	1.12 E−05	Down	trypsin, alkaline C-like [Spodoptera litura]	PRSS1_2_3
				TRINITY_DN13619_c0_g2_i1_3	701.0350489	199.5322761	0.28462525	0.00871419	0.3479086	Down	sodium/potassium-transporting ATPase subunit alpha isoform X6 [Bombyx mori]	ATP1A
				TRINITY_DN13597_c0_g2_i2_4	683.1424229	36.96948382	0.054116803	0.03730582	0.7023823	Down	serine protease 62 [Mamestra configurata]	PRSS1_2_3
				TRINITY_DN10836_c0_g7_i1_6	407.2114124	52.04490715	0.127808076	0.00142694	0.1232753	Down	trypsin, partial [Manduca sexta]	PRSS1_2_3
				TRINITY_DN7116_c0_g1_i1_5	140.0397486	41.73985284	0.298057182	0.02906	0.6296715	Down	Prolylcarboxypeptidase [Danaus plexippus plexippus]	PRCP
				TRINITY_DN5681_c0_g1_i1_6	1202.562374	41.63057875	0.034618228	0.00013096	0.0240473	Down	chymotrypsinogen-like protein 3 [Manduca sexta]	PRSS1_2_3
				TRINITY_DN10836_c0_g5_i1_6	3539.814633	11.52410727	0.003255568	0.00393418	0.2234223	Down	trypsin, alkaline C [Bombyx mori]	PRSS1_2_3
				TRINITY_DN14237_c1_g1_i3_3	2139.270665	126.8457966	0.059293945	0.00362815	0.2142243	Down	hypothetical protein B5V51_4161 [Heliothis virescens]	PRSS1_2_3
				TRINITY_DN18044_c0_g1_i1_4	61.20950346	0	0	0.00262208	0.1786659	Down	RecName: Full = Trypsin, alkaline C; Flags: Precursor	PRSS1_2_3
				TRINITY_DN13619_c0_g3_i1_3	1098.504638	384.681457	0.350186466	0.02344348	0.5771173	Down	Sodium/potassium-transporting ATPase subunit alpha [Papilio xuthus]	ATP1A
				TRINITY_DN12770_c1_g2_i2_6	33,988.65545	2472.057397	0.072731838	0.03984473	0.7217322	Down	serine protease 62 [Mamestra configurata]	PRSS1_2_3
				TRINITY_DN14161_c2_g2_i3_3	150,880.9838	10,699.40148	0.070912856	0.027439	0.6151748	Down	trypsin, alkaline C-like [Spodoptera litura]	PRSS1_2_3
				TRINITY_DN12929_c2_g1_i1_6	9158.048497	41.0899202	0.004486755	0.00143302	0.1232753	Down	trypsin [Manduca sexta]	PRSS1_2_3
				TRINITY_DN10836_c0_g1_i4_6	157,887.1736	53,448.64932	0.338524328	0.02226533	0.5641697	Down	trypsin, alkaline C-like [Bombyx mori]	PRSS1_2_3
				TRINITY_DN12646_c0_g1_i3_6	18,799.76899	343.0033323	0.018245082	0.00558226	0.271197	Down	trypsin, alkaline C-like [Spodoptera litura]	PRSS1_2_3
				TRINITY_DN3826_c0_g1_i1_3	4708.243184	116.815754	0.024810901	0.00083317	0.0853474	Down	serine protease 5 [Mamestra configurata]	PRSS1_2_3
				TRINITY_DN12903_c0_g1_i1_6	588.272948	19.69837276	0.03348509	7.39 E−10	8.85 E−07	Down	silk gland derived serine protease [Bombyx mori]	PRSS1_2_3
				TRINITY_DN14269_c4_g1_i5_4	15,015.14212	207.7199418	0.013834031	0.00010037	0.019782	Down	trypsin [Manduca sexta]	PRSS1_2_3
				TRINITY_DN8384_c0_g2_i8_4	4501.305649	35.57861617	0.007904066	0.00033725	0.0475214	Down	chymotrypsinogen-like protein 3 [Manduca sexta]	PRSS1_2_3
				TRINITY_DN17232_c0_g1_i1_5	229.0935699	80.06529495	0.349487308	0.03584292	0.6919927	Down	proton-coupled amino acid transporter-like protein CG1139 [Trichoplusia ni]	SLC36A, PAT
				TRINITY_DN8969_c0_g1_i1_6	1070.195299	24.35172941	0.022754472	0.00153816	0.1284174	Down	carboxypeptidase B [Bombyx mori]	CPA2
				TRINITY_DN12498_c2_g2_i1_5	3577.880426	0	0	0.00308839	0.197654	Down	trypsin CFT-1-like [Trichoplusia ni]	PRSS1_2_3
				TRINITY_DN9363_c0_g1_i1_5	2166.100895	67.76280065	0.031283308	7.81 E−11	1.52 E−07	Down	trypsin precursor AiD2, partial [Agrotis ipsilon]	PRSS1_2_3
				TRINITY_DN7771_c0_g1_i1_1	166.8439433	24.32627007	0.145802536	0.01850363	0.5173478	Down	hypothetical protein B5V51_4161 [Heliothis virescens]	PRSS1_2_3
				TRINITY_DN1220_c0_g1_i1_5	14,636.6041	1623.956772	0.110951745	6.30 E−06	0.0020452	Down	trypsin, alkaline C-like [Spodoptera litura]	PRSS1_2_3
ko04977	Vitamin digestion and absorption	8.59 E−05	4.753950617	TRINITY_DN8071_c0_g1_i2_5	159.1367963	7.893169411	0.049599901	2.35 E−05	0.0062453	Down	proton-coupled folate transporter isoform X2 [Bombyx mori]	SLC46A1
				TRINITY_DN12381_c0_g2_i1_6	15,115.15447	69.82405532	0.004619473	1.51 E−05	0.0042801	Down	pancreatic triacylglycerol lipas E−like [Spodoptera litura]	PNLIP, PL
				TRINITY_DN9781_c0_g1_i1_3	75.2460337	20.63729453	0.274264217	0.03100164	0.6531949	Down	scavenger receptor class B type 1 like protein 12 [Bombyx mori]	SCARB1
				TRINITY_DN11521_c0_g1_i2_4	1196.401288	280.0407483	0.234069247	0.00250876	0.1751687	Down	solute carrier family 52, riboflavin transporter, member 3-B isoform X3 [Trichoplusia ni]	SLC52A3, RFT2
				TRINITY_DN14080_c0_g1_i4_5	13,236.92182	908.4409062	0.068629317	0.03090689	0.6516395	Down	pancreatic triacylglycerol lipase [Bombyx mori]	PNLIP, PL
				TRINITY_DN17108_c0_g1_i1_5	19.89691746	1.317177118	0.066200059	0.0121168	0.4110339	Down	hypothetical protein B5V51_177 [Heliothis virescens]	SLC46A1
				TRINITY_DN14140_c0_g1_i1_6	6399.436654	1095.005085	0.171109606	0.00016917	0.0286312	Down	sensory neuron membrane protein 2 [Bombyx mori]	SCARB1
ko04624	Toll and Imd signaling pathway	0.00016	3.943369176	TRINITY_DN13195_c0_g1_i3_3	48,818.06612	740.1059156	0.015160492	2.79 E−06	0.0010722	Down	peptidoglycan recognition protein 2 [Manduca sexta]	PGRP
				TRINITY_DN1052_c0_g1_i2_5	74.58752535	0	0	0.02832612	0.6208957	Down	Bacteriophage T7 lysozym E−like protein 1 (BTL-LP1) [Bombyx mori]	PGRP
				TRINITY_DN10280_c0_g1_i1_3	1415.480197	536.8363029	0.379260907	0.04150037	0.7315422	Down	caspas E−6 [Manduca sexta]	CASP8
				TRINITY_DN14006_c2_g1_i2_4	16,714.28346	318.7737419	0.019071936	3.82 E−10	5.17 E−07	Down	peptidoglycan recognition protein 2 [Manduca sexta]	PGRP
ko00830	Retinol metabolism	0.000409	3.492698413	TRINITY_DN14190_c1_g2_i2_4	4018.349256	720.63212	0.179335362	0.04158394	0.7316189	Down	UDP-glucosyltransferase isoform X1 [Bombyx mori]	UGT
				TRINITY_DN12319_c0_g2_i1_4	4465.699274	170.972127	0.038285634	2.79 E−06	0.0010722	Down	UDP-glycosyltransferase UGT340C2 [Bombyx mori]	UGT
				TRINITY_DN12896_c1_g2_i3_3	4251.17249	1508.008769	0.35472773	0.02274633	0.5685456	Down	PREDICTED: RNA-directed DNA polymerase from mobile element jockey-like [Papilio machaon]	DHRS4
				TRINITY_DN13518_c1_g1_i6_6	745.6825793	119.2237632	0.159885408	0.0001628	0.0278557	Down	UDP-glycosyltransferase UGT340C1 precursor [Bombyx mori]	UGT
				TRINITY_DN14445_c0_g1_i1_3	151.0781746	54.27468401	0.359249006	0.04671659	0.7685163	Down	hypothetical protein B5X24_HaOG201493 [Helicoverpa armigera]	RDH12
				TRINITY_DN9738_c0_g1_i1_6	438.41149	83.17535239	0.189719828	0.04256225	0.7412925	Down	uncharacterized protein LOC112052352 [Bicyclus anynana]	UGT
				TRINITY_DN8673_c0_g1_i3_3	839.7824168	167.2848772	0.199200262	0.00073535	0.0803495	Down	PREDICTED: UDP-glucuronosyltransferase 2B19-like isoform X6 [Amyelois transitella]	UGT
				TRINITY_DN17220_c0_g1_i1_4	6379.593263	3.929259199	0.000615911	0.00570705	0.2744919	Down	UDP-glycosyltransferase UGT340C1 precursor [Bombyx mori]	UGT

In this study, the up-regulation of glycerophospholipid metabolism was consistent with Zhang’s research [21]. The up-regulation of this pathway may be related to the viral replication [39, 40]. In addition, Glycine, serine and threonine metabolism were up-regulated in this transcriptome analysis. In the study by Wu et al., two genes related to this signaling pathway were up-regulated and the other down-regulated. In our study, the expression levels of the phosphoserine phosphatase genes were significantly higher in DnCPV-23-infected midgut than in the non-infected group, suggesting that serine metabolism disorders were induced after DnCPV-23 infection. Expression of many *UGT* genes was up-regulated; UDP-glucuronosyltransferase (UGT) isozymes take endogenic and exogenic toxic substances as substrates, catalyze detoxification of many chemical toxins in our daily diet and environment by conjugation to glucuronic acid or glucose [41, 42]. After DnCPV-23 infection, it was speculated that the *D. nerii* tended to strengthen the elimination of lipophilic endobiotics such as hormones and xenobiotics including phytoalexins and drugs conjugated by invertebrates and plants mainly with glucose [42] through promoting the transcription of *UGTs* by regulating the activities of nuclear-receptor family (CAR, PXR, FXR, LXR, and PPAR), the arylhydrocarbon receptor [43] or ubiquitous transcription factors (FOXA1, Sp1, and Cdx2) [44]. However, the interactions between UGT and cypovirus still remain unclear. In Table 5, there were the pathways and genes mentioned above and genes expression of each sample is shown in Additional file 1.

**Table 5:** The up-regulated pathways focused in the discussion section

id	Term	pValue	Enrichment_score	Gene_id	BaseMean_control_mock	BaseMean_case_DnCPV	FoldChange	pValue	qValue	Regulation	NR annotation	KEGG gene name
ko00564	Glycerophospholipid metabolism	0.00046	3.794540796	TRINITY_DN14020_c0_g1_i1_6	1066.209777	3311.867512	3.106206287	0.027085	0.610813421	Up	phosphatidate phosphatase LPIN2 isoform X2 [Trichoplusia ni]	LPIN
				TRINITY_DN14343_c0_g2_i1_4	25.87214477	100.8782352	3.899106012	0.02501	0.591372647	Up	hypothetical protein B5V51_748 [Heliothis virescens]	NTE, NRE
				TRINITY_DN14343_c2_g1_i1_5	1212.926019	3816.360113	3.146407986	0.018214	0.514696311	Up	phosphatidate phosphatase LPIN3 isoform X1 [Bombyx mori]	LPIN
				TRINITY_DN2180_c0_g1_i1_3	5.852454693	49.91148246	8.528298822	0.005837	0.276535057	Up	group XV phospholipase A2-like [Trichoplusia ni]	LYPLA3
				TRINITY_DN10250_c0_g1_i1_1	73.45139125	221.6966763	3.018277429	0.037654	0.703084732	Up	group XV phospholipase A2-like [Trichoplusia ni]	LYPLA3
				TRINITY_DN11518_c6_g1_i1_2	52.98336083	709.749683	13.39570899	0.008188	0.337015221	Up	Phosphatidylserine decarboxylase [Operophtera brumata]	psd, PISD
				TRINITY_DN12265_c0_g2_i1_2	21.24218102	111.7071749	5.258743197	0.006687	0.296214335	Up	Neuropathy target esterase sws [Papilio xuthus]	NTE, NRE
ko00260	Glycine, serine and threonine metabolism	0.00232	4.238058552	TRINITY_DN9933_c0_g1_i2_6	782.5009178	4185.641092	5.349055824	0.025238	0.593696583	Up	phosphoserine phosphatase isoform X3 [Trichoplusia ni]	serB, PSPH
				TRINITY_DN7804_c0_g1_i1_2	11.1084935	91.01956837	8.193691462	0.032267	0.660850357	Up	glucose dehydrogenase [FAD, quinone] [Bombyx mori]	betA, CHDH
				TRINITY_DN12220_c1_g1_i9_4	107.649078	517.8794976	4.8108122	0.00313	0.19793455	Up	PREDICTED: phosphoserine phosphatase [Amyelois transitella]	serB, PSPH
				TRINITY_DN10934_c0_g2_i2_1	2279.078944	10,414.52625	4.569620669	0.002617	0.17866588	Up	phosphoserine phosphatase isoform X1 [Bombyx mori]	serB, PSPH
ko00982	Drug metabolism—cytochrome P450	0.0002	4.29382248	TRINITY_DN11538_c1_g1_i3_2	228.6892992	2097.050761	9.169868326	0.023489	0.577337157	Up	hypothetical protein B5V51_11710 [Heliothis virescens]	UGT
				TRINITY_DN14215_c0_g5_i7_5	127.0025656	13,180.97268	103.7850898	0.020886	0.5511394	Up	UDP-glucuronosyltransferase 1-7C-like [Trichoplusia ni]	UGT
				TRINITY_DN7938_c0_g2_i1_2	3.93470221	637.5929402	162.0435058	6.29 E−05	0.013882511	Up	PREDICTED: uncharacterized protein LOC106102769 [Papilio polytes]	GST, gst
				TRINITY_DN13727_c0_g2_i1_5	185.9951388	4205.660038	22.61166644	0.002663	0.180041496	Up	UDP-glycosyltransferase UGT340C2 [Bombyx mori]	UGT
				TRINITY_DN13616_c0_g3_i6_5	25.95433532	8843.383538	340.7285692	0.014481	0.449089688	Up	UDP-glucuronosyltransferase 1-7C-like [Trichoplusia ni]	UGT
				TRINITY_DN11622_c2_g4_i1_2	0	23.44067936	Inf	0.026206	0.601380717	Up	UDP-glucuronosyltransferase 2B15-like isoform X1 [Helicoverpa armigera]	UGT
				TRINITY_DN11402_c0_g2_i13_2	663.9094166	6959.047076	10.48192254	0.000742	0.080554531	Up	UDP-glucuronosyltransferase 1-7C-like [Trichoplusia ni]	UGT
ko00980	Metabolism of xenobiotics by cytochrome P450	0.00043	3.839182453	TRINITY_DN11538_c1_g1_i3_2	228.6892992	2097.050761	9.169868326	0.023489	0.577337157	Up	hypothetical protein B5V51_11710 [Heliothis virescens]	UGT
				TRINITY_DN14215_c0_g5_i7_5	127.0025656	13,180.97268	103.7850898	0.020886	0.5511394	Up	UDP-glucuronosyltransferase 1-7C-like [Trichoplusia ni]	UGT
				TRINITY_DN7938_c0_g2_i1_2	3.93470221	637.5929402	162.0435058	6.29 E− E−05	0.013882511	Up	PREDICTED: uncharacterized protein LOC106102769 [Papilio polytes]	GST, gst
				TRINITY_DN13727_c0_g2_i1_5	185.9951388	4205.660038	22.61166644	0.002663	0.180041496	Up	UDP-glycosyltransferase UGT340C2 [Bombyx mori]	UGT
				TRINITY_DN13616_c0_g3_i6_5	25.95433532	8843.383538	340.7285692	0.014481	0.449089688	Up	UDP-glucuronosyltransferase 1-7C-like [Trichoplusia ni]	UGT
				TRINITY_DN11622_c2_g4_i1_2	0	23.44067936	Inf	0.026206	0.601380717	Up	UDP-glucuronosyltransferase 2B15-like isoform X1 [Helicoverpa armigera]	UGT
				TRINITY_DN11402_c0_g2_i13_2	663.9094166	6959.047076	10.48192254	0.000742	0.080554531	Up	UDP-glucuronosyltransferase 1-7C-like [Trichoplusia ni]	UGT
ko00983	Drug metabolism—other enzymes	0.00101	3.107909605	TRINITY_DN11538_c1_g1_i3_2	228.6892992	2097.050761	9.169868326	0.023489	0.577337157	Up	hypothetical protein B5V51_11710 [Heliothis virescens]	UGT
				TRINITY_DN14215_c0_g5_i7_5	127.0025656	13,180.97268	103.7850898	0.020886	0.5511394	Up	UDP-glucuronosyltransferase 1-7C-like [Trichoplusia ni]	UGT
				TRINITY_DN7938_c0_g2_i1_2	3.93470221	637.5929402	162.0435058	6.29 E−05	0.013882511	Up	PREDICTED: uncharacterized protein LOC106102769 [Papilio polytes]	GST, gst
				TRINITY_DN13727_c0_g2_i1_5	185.9951388	4205.660038	22.61166644	0.002663	0.180041496	Up	UDP-glycosyltransferase UGT340C2 [Bombyx mori]	UGT
				TRINITY_DN11728_c0_g1_i4_2	1306.667581	6467.759766	4.949812684	0.001549	0.128973078	Up	uridine phosphorylase 1 isoform X2 [Bombyx mori]	udp, UPP
				TRINITY_DN13616_c0_g3_i6_5	25.95433532	8843.383538	340.7285692	0.014481	0.449089688	Up	UDP-glucuronosyltransferase 1-7C-like [Trichoplusia ni]	UGT
				TRINITY_DN11622_c2_g4_i1_2	0	23.44067936	Inf	0.026206	0.601380717	Up	UDP-glucuronosyltransferase 2B15-like isoform X1 [Helicoverpa armigera]	UGT
				TRINITY_DN11402_c0_g2_i13_2	663.9094166	6959.047076	10.48192254	0.000742	0.080554531	Up	UDP-glucuronosyltransferase 1-7C-like [Trichoplusia ni]	UGT

## Conclusion

This study revealed substantial differences in the transcriptions of the *D. nerii* genes related to digestion, immunity, glycerophospholipid metabolism and toxic substances metabolism induced by DnCPV-23 replication. Findings obtained in this research further enriched the understanding of cypovirus-*Spodoptera* insect interactions in midgut and provided additional basic information for the future exploitation of DnCPV-23.

## Supplementary Information

**Additional file 1**. All the different expression genes in the midgut after DnCPV-23 infection.

## Data Availability

The original data of the transcriptome will be released on 2021-10-05 or upon publicationhas, BioProject accession: PRJNA766516.

## Abbreviations

DnCPV-23: *Daphnis nerii* Cypovirus-23
*D.** nerii*: *Daphnis nerii*
PGRP: Peptidoglycan Recognition Protein;
CASP-8: Caspase-8
